# OMICS Approaches Evaluating Keloid and Hypertrophic Scars

**DOI:** 10.1155/2022/1490492

**Published:** 2022-10-27

**Authors:** Nazihah Bakhtyar, Saeid Amini-Nik, Marc G. Jeschke

**Affiliations:** ^1^Sunnybrook Health Sciences Center, Sunnybrook Research Institute, Toronto, Ontario, Canada; ^2^Sunnybrook Health Sciences Center, Sunnybrook Research Institute, Department of Laboratory Medicine and Pathobiology (LMP), The University of Toronto, Toronto, Ontario, Canada; ^3^Health Sciences Center, Sunnybrook Research Institute, Ross Tilley Burn Center, Sunnybrook's Trauma, Emergency & Critical Care (TECC) Program, The University of Toronto, Toronto, Ontario, Canada

## Abstract

Abnormal scar formation during wound healing can result in keloid and hypertrophic scars, which is a major global health challenge. Such abnormal scars can cause significant physiological pain and psychological distress and become a financial burden. Due to the biological complexity of scar formation, the pathogenesis of such scars and how to prevent them from forming remains elusive. In this review paper, we delve into the world of “omics” approaches to study abnormal scars and provide examples of genomics, transcriptomics, proteomics, epigenomics, and metabolomics. The benefits of “omics” approaches are that they allow for high-throughput studies and the analysis of 100s to 1000s of genes and proteins with the accumulation of large quantities of data. Currently in the field, there is a lack of “omics” review articles describing pathological scars. In this review, we summarize genome-wide linkage analysis, genome-wide association studies, and microarray data to name a few omics technologies. Such data can provide novel insights into different molecular pathways and identify novel factors which may not be captured through small-scale laboratory techniques.

## 1. Introduction

### 1.1. The Key Phases of Wound Healing

Skin wound healing is a complex process which encompasses three coordinated phases. These phases of wound healing are the inflammatory, proliferative, and remodeling phases [1]. When a wound occurs, blood leaks from damaged blood vessels into the surrounding tissue, and this must be prevented to halt further blood and fluid loss. The initial inflammatory phase, therefore, starts with a hemostasis step which involves the blood coagulation cascade. A platelet plug forms to prevent further blood loss and allows cells to migrate during wound repair. Fibrin forms as a result of thrombin cleavage of fibrinogen with the addition of plasma fibronectin, vitronectin, and thrombospondin, which creates a mesh-like structure. In addition to clot formation preventing further blood loss, the clot serves a second very important function; the clot platelets release cytokines and growth factors. These cytokines and growth factors recruit inflammatory cells [1, 2].

Inflammatory pathways and the immune system participate in wound healing to rid the wound of dead or dying tissues and to prevent infections [3, 4]. Stimulation of the complement cascade leads to the recruitment of neutrophils, which clean the wound of bacteria and tissue debris. Furthermore, neutrophils release fibronectin, which has multiple roles; these include serving a structural role because of its fibrillary makeup, assisting with interactions between the ECM and various other cells or acting as a bridge amongst cells [2, 5].

Next, the proliferative phase occurs where there is reepithelialization of the epidermis and repair of the underlying dermis tissue by infiltration and proliferation of fibroblasts and keratinocytes with concurrent angiogenesis and formation of *de novo* blood vessels [3, 4]. Macrophages begin this process by breaking the blood clots and producing an array of chemokines and cytokines, such as, TGF-*α*, TGF-*β*, interleukin 1, and insulin-like growth factor I. These act as chemotactic signals necessary to attract fibroblasts into the wound site in order to initiate wound closure [3, 6]. Epidermal cells at the wound edge undergo phenotypic changes, such as keratinocyte activation to be able to migrate over the wound bed. The dermis is reestablished by fibroblasts which migrate and proliferate in the wound. The fibroblasts are able to migrate into the wound as a result of secretion of extracellular matrix (ECM)-cleaving matrix metalloproteinases (MMP), which cause ECM breakdown [2]. TGF-*β*s are instrumental in regulating the development of the ECM [7]. The ECM is also composed of glycoproteins, collagens, glycosaminoglycans, and proteoglycans. Proteoglycans take part in cell to cell and cell with matrix interactions, proliferation of cells, and migration, in addition to cytokine and growth factor signaling related to wound healing. Macrophages produce reactive oxygen species (ROS) and MMPs that then enhance keratinocyte proliferation and migration [8]. In order for a wound to heal, there must be a supply of oxygen through new blood capillaries to the wound bed. The hypoxic condition of a wound stimulates macrophages, keratinocytes, fibroblasts, and endothelial cells to generate more vascular endothelial growth factor (VEGF), a cytokine, which plays an important role in angiogenesis [6].

The production of collagenase by epidermal cells and the activation of plasmin by plasminogen activator is necessary for the further breakdown of the ECM and for the migration of cells [9]. Behind these migrating cells, epidermal cells start proliferating. Fibroblasts, which are mainly attracted to the wound edge are stimulated by macrophages to differentiate into myofibroblasts [10–12]. Myofibroblasts in turn help pull the wound edges together, leading to faster contraction, which then leads to wound healing [11–13]. Fibroblasts and myofibroblasts produce ECM, primarily collagen, which forms the structural framework of granulation tissue [4, 14].

The last stage of wound healing is remodeling. This stage is driven by collagen remodeling, which allows the granulation tissue to become a scar. This process depends on the continuous synthesis and breakdown of collagen/ECM. Although there is an increase in both collagen types I and III in an immature scar, more specifically, there is the replacement of collagen type III by collagen type I. The disintegration of collagen in the wound is managed by various proteolytic enzymes such as matrix metalloproteinases that are secreted by macrophages, epidermal cells, endothelial cells, and fibroblasts [2]. Furthermore, there is apoptosis of various cells within the wound. Alogether, this results in wound contraction and the reconstitution of damaged skin, which is associated with scar formation in humans. At the completion of wound healing, myofibroblasts undergo apoptosis, and the failure of these cells to undergo apoptosis often leads to pathological scarring [12, 15]. The proteins participating in re-epithelialization include multiple extracellular-matrix proteins and their receptors, proteases, cytoskeletal proteins, and enzymes that modulate the cellular redox balance [4]. Gurtner et al have also shown that cytoskeletal proteins play a significant role in mechanical tension, which upregulates fibrotic pathways in humans and lead to more scar tissue formation [4].

### 1.2. Abnormal Wound Healing Resulting in Keloid and HTS

If the steps in wound healing do not occur in a synchronized manner, pathologic or abnormal wound healing results. Improper ECM reorganisation, which includes a disequilibrium of the breakdown and buildup of ECM, can lead to excess scarring. A prolonged inflammatory phase is believed to initiate abnormal wound healing. Some examples of excessive wound healing include fibrosis, strictures, adhesions, and contractures. Keloids and hypertrophic scars (HTS) are examples of fibroproliferative disorders and are characterized by the excess buildup of collagen within the wound [16, 17]. Scars may form after injury to the deep dermis. Examples of such injuries include burns, cuts, abrasions, piercings, vaccinations, and surgery [18].

#### 1.2.1. Keloid

The first report on excessive scar tissue formation was documented in the Smith papyrus around 1700 BC [19]. In 1806, Alibert first discussed this scar tissue; he thought it resembled a tumor and therefore called it a cancroid, but later changed the term to cheloide. Cheloide comes from the Greek term chele, which means crab claw, and describes how the scar grows laterally into the normal tissue [19].

Keloids may develop after minor injuries to different parts of the body or even without any injury to the sternum [20, 21]. Keloid formation can occur many years after even the minor injuries to the dermal tissue, and they can persist for an extended period of time and do not regress spontaneously [22]. Keloids usually grow beyond the confines of their initial injury, and their size may remain the same, or they may grow with time [22]. An important determinant of keloid formation is wound tension. One will often find keloids forming in parts of the body where there is high tension; this includes regions such as, upper back, shoulders, cheeks, anterior chest, and upper arms [22]. Upon examination, keloids look firm, they are raised and tumor-like with a shiny surface. The epithelium is thin, and sometimes there is a central ulcer. The color of a keloid is pinkish purple and could have hyperpigmentation [18, 23]. Keloids have high levels of fibroblast proliferation rates, and their collagen fibers are larger and thicker and consist of a more wavy and disorganized pattern compared to hypertrophic or normal scars [24, 25]. They are pruritic and can also cause significant pain and hyperesthesia [18].

#### 1.2.2. Hypertrophic Scars

In 1962, Mancini and in 1970, Peacock described excess scar tissue as hypertrophic if it rises above the skin but stays within the boundaries of the wound [26, 27]. Hypertrophic scars are mostly formed after burns or trauma to the deep dermis; they appear as red, raised, and in a linear scar pattern which can appear in any part of the body. Some specific areas of the body where HTS scars predominate are shoulders, neck, presternum, knees, and ankles [20, 28]. Hypertrophic scarring is often initiated within one to two months after injuries to the body, such as, skin injury after wound infection or wound closure with tension in the skin. It should also be noted that HTSs have a different growth pattern than keloid scars, although sometimes keloids and HTSs are difficult to differentiate. Unlike keloids, HTS grows rapidly for up to 6 months and then regresses over the next few years and finally resulting in flattened scars with no further symptoms. References [18, 21].

In terms of the composition of the HTS, they are described as having a significant amount of alpha-smooth muscle actin (*α*-SMA) producing myofibroblasts. In addition, there is a greater quantity of type III collagen than type I collagen. This is different from keloids, because keloids have a mixture of both type I and type III collagen [29]. The collagen bundles in HTS are fine, in an organized pattern and parallel to the epidermis. In comparison to keloids, HTS treatment has a better prognosis after surgical excision because HTS has lower recurrence rates [18]. Complications from this type of scar include pain, pruritus, immobility of joints, and disfigurement [21].

## 2. Demographics and Pathophysiology

### 2.1. Sex/Gender

Several reports suggest that keloid and HTS formation are equal in incidence among the two sexes. The highest incidence occurs between the ages of 10 and 30 [18, 30]. However, a report by Bayat et al. showed that there was a correlation between female sex and the formation of keloid masses in multiple areas. Furthermore, the frequency of all keloid scars was higher in females compared to males except in the face, the posterior auricular area, and the scalp. Female patients with familial keloids have a higher frequency of getting keloids compared to male patients who also have a family history of keloids [31]. Reports on HTS formation do not show any predominance amongst females compared to males, reports on HTS describe an equal occurrence of the scar in both sexes [18].

### 2.2. Race/Ethnicity

#### 2.2.1. Keloids

The prevalence of keloid formation is different based on the population being studied. Epidemiological data suggest that keloid formation varies based on race, with higher rates occurring in Blacks, Hispanics, and Asians [19, 32, 33]. The highest incidence of keloids is in the black or African population, ranging from 6–16% [18, 19, 33, 34]. Individuals with a darker phenotype tend to have a higher risk of keloid development [35, 36]. Caucasian and albino individuals are the least affected by keloids, highlighting a correlation between ethnicity and keloid formation. This suggests that there might be a genetic component to keloid formation [19, 32].

#### 2.2.2. HTS

Hypertrophic scars form after surgery and burns, with an incidence ranging from 40%–70% following surgery and up to 91% following burn injury [18]. There has not been any correlation linking hypertrophic scars to ethnicity as there is with keloid formation [18].

### 2.3. Pathophysiology of Keloids and HTS

As stated earlier, a disequilibrium between the breakdown and buildup of ECM can lead to excess scarring. Collagen plays a critical role. In keloids, the collagen synthesis may be up to 20 times as high as the collagen synthesis in normal wound healing scars. This exacerbated collagen production is related to the highly proliferative fibroblasts of the keloid [37, 38]. The rate of collagen synthesis is less pronounced in HTS, where it is three times as high as in normal unscarred skin [38]. Within the different types of collagen, the ratio of type I to type III is deemed to be higher [39]. Certain growth factors are also involved in keloids and HTS, such as TGF- *β*, PDGF, and IGF. As described in the introduction to this review, TGF- *β* is instrumental to normal wound healing for the chemotaxis of fibroblasts, fibroblast proliferation and collagen production. In keloids, TGF-*β* is hyperproduced and unable to be regulated by normal physiological autocrine signals [40]. Furthermore, keloid fibroblasts tend to have a higher number of receptors for growth factors and therefore, are more responsive to TGF-b and PDGF [40]. An increase in IGF-1 leads to the expression of types I and III procollagen and the IGF-1 receptor was demonstrated to be overexpressed in keloid fibroblasts [41]. It has also been reported that dysregulation of certain cytokines, including interleukins 6, 13, and 15 may be involved in keloid scarring [42]. Some other factors involved in keloid and HTS formation have been reported to be the skin's distorted cellular immune response [43], tissue hypoxia [44], abnormal fatty acid configuration of the fibroblasts, elevated levels of nitric oxide throughout wound healing [45], and perhaps an immune response to sebum [46]. Further research is warranted and a deep look into the omics of scarring will help elucidate some of these pathophysiological mysteries.

## 3. Genomics of Keloids

### 3.1. Family History and Inheritance Patterns

Patients with keloids often demonstrate familial keloid formation, suggesting a genetic predisposition to this form of abnormal scarring. In a study by Marneros et al., they looked at 14 pedigrees with keloids in the family, and the pedigrees spanned at least 3 generations. Pedigree charts were used to analyze the mode of keloid inheritance [47]. The total number of family members were 341 with 96 who had keloid manifestation. The pattern of inheritance was autosomal dominant with incomplete clinical penetrance and variable expression. This was deduced through pedigree analysis. The results examining affected and unaffected children and whether or not the parents had keloids established that nonpenetrance accounts for 6.8% of the individuals. They were therefore obligate unaffected carriers in their families. The data from this paper shows that there is keloid manifestation in different populations because the analysis was conducted on families from different ethnicities. The ethnicities of these families were majority African American (*n* = 10), Caucasian (*n* = 1), Japanese (*n* = 2), and African Caribbean (*n* = 1) [39]. The analysis revealed different disease onsets, variable expression, and different responses to treatments. It should be noted that this study does not investigate different genes which could be involved in familial keloid formation but focuses on clinical genetic characteristics.

Omo-Dare studied a group of 75 people for 3 generations in a west Nigerian town called Igbo-Ora in order to investigate the role played by inheritance in keloid formation. For this effort, pedigree charts were created. Results illustrate that the rate of affected offspring of mothers who had keloids was 11/49 (22.45%), which does not support the dominant mode of inheritance pattern. In this study, two matings between people who were both keloid-forming produced two keloid-forming offspring. Twenty nonkeloid with nonkeloid forming matings produced some offspring with keloids. This data suggest that keloid formation occurs in a recessive pattern. The analysis revealed that the 22.45% rate of keloid formation in offspring is a Mendelian ratio for recessive inheritance and does not agree with dominant gene inheritance proportions Omo-Dare et al. also concluded that since there was no difference between sex, keloid inheritance was autosomal [34]. In this paper, the authors are not suggesting that keloid formation is multifactorial because for multifactorial inheritance, there cannot be dominance. The prevalence distribution curve of keloid formation for this study is discontinuous, suggesting that keloid character is determined by two genes, one of which is dominant to the other. Once again, it should be noted that this paper does not investigate specific genes.

Chen et al. studied keloid inheritance in the Han Chinese population. They investigated 6 pedigrees. The pedigrees include a total of 185 family members, 94 were male and 91 were female. Pedigree analysis displayed an autosomal dominant mode of inheritance with incomplete clinical penetrance. Having incomplete penetrance means that carrying the dominant allele does not guarantee the expression of the phenotype. Chen et al. also reported that Chinese familial keloids occur during adolescence, which is a time of high hormone secretion, causing one to speculate whether there is a hormonal factor involved in keloid formation [48]. Of the 185 family members in the pedigrees, 45 displayed keloids, and of the impacted members of the family, there were 18 male and 27 female [48]. Three pedigrees encompass 4 generations; two pedigrees span 3 generations; and one pedigree was for 5 generations. One pedigree displayed keloids in all 5 generations; two pedigrees displayed in all 4 generations; two displayed in 2 generations out of a total of 3 generations; and one in 3 generations out of 4 total generations studied. For this study, the main factor causing keloids was acne. From all of these pedigrees, five of the family members were described as obligate unaffected carriers, and 45 members were affected with keloids, revealing that nonpenetrance comprises 10% of keloid gene carriers. These data provide evidence for the pattern of inheritance in the Han Chinese families to be in accordance with an autosomal dominant mode with incomplete clinical penetrance. Nonpenetrance more frequently takes place under dominant conditions [48]. The different types of inheritance patterns are summarized in Table 1. It has been reported that androgen in keloids is elevated compared to neighboring normal skin. Androgen was also 10-fold higher in keloid scars as opposed to normal scars in a report by Ford et al. [49]. The literature suggests that keloid formation is high during puberty because of high levels of hormones [37, 50]. This hormone hypothesis could also be a reason for the incomplete penetrance observed in many pedigree studies. It should be noted that Bayat et al. report that females develop keloids more frequently than males, but their reasoning is specific to the ear lobe anatomical region, suggesting that females undergo more ear piercings, which enhance the risk of developing keloids [51].

### 3.2. Linkage Studies

After Marneros et al. established that keloids form in an autosomal dominant manner in families by studying pedigrees, they aimed to identify genetic susceptibility loci. They performed a genome-wide linkage search in two families, one Japanese and the other African American. They explored genes predisposing to keloid formation. A genome-wide linkage study for the Japanese family demonstrated a two-point LOD (logarithm of odds) scores >2 for a chromosomal region on 2q23. Marker D2S1399 (152 cM; 148 Mbp) showed a maximum two-point LOD score of 3.01. Haplotype assessment revealed a disease allele for all impacted members within the family in a 40 cM interval between D2S410 and D2S1353. This locus has genes that are involved in regulating wound healing responses and were thus considered to be candidate genes for hereditary keloids. For example, the TNF-a inhibitory protein 6, TNFAIP6, was mapped near the chromosome 2q23 marker which displayed the high LOD score for this Japanese family. For the African American family, genome-wide linkage analysis recognized one chromosomal region with two-point LOD scores >2. On chromosome 7p11, marker D7S3046 (79 cM; 68 Mbp) uncovered a two-point LOD score of 2.72. Marker D7S499 on chromosome 7p11 demonstrated the highest two-point LOD score of 3.16. Marker D7S499 is associated with the EGF receptor gene [52]. These findings are summarized in Table 2.

Yan et al. studied a Chinese family for five generations, utilizing seven markers to study two loci at chromosome 15q22.31-q23 and 18q21.1 [53]. Seven microsatellite markers covering key domains on chromosomes 15q22.31-q23 and 18q21.1 were genotyped. Next, the DNA obtained from each sample was expanded to examine these markers. A maximum LOD score of 2.201 was seen at 18q21.1 suggesting that this locus could be a predisposition locus, spanning the genes SMAD2, SMAD4, SMAD7, and PLAS2. SMAD genes play a role in regulating the TGF-*β* pathway; TGF-*β* pathway has been highly investigated to be involved in fibrotic diseases and keloid development [53]. Chen et al. also studied the Han Chinese family keloid pedigree for five generations [54]. Since the FAS gene was understood to be one of the candidate genes causing keloids for this pedigree, four markers besides all the known genes relating to apoptosis or tumorigenesis in the 10 Mbp region around the FAS gene on chromosomes 10q23.31 were identified as microsatellite markers [54]. They provided genetic evidence that in the Han Chinese keloid family pedigree, there was a predisposing gene which could be represented by an area of about 1 Mbp on chromosome 10q23.31, between markers D10S1765 and D10S1735 [54]. These results are displayed in Table 2.

### 3.3. Gene Association

There is substantial amount of evidence which points to TGF-*β* as a major cytokine which commences and terminates tissue repair. Overproduction of TGF-*β* could potentially cause the development of fibrosis with excess deposition of scar tissue. TGF-*β* is said to induce ECM components [55]. Therefore TGF-*β* has been associated with the pathogenesis of keloid disease and HTS. In a study by Bayat et al., they aimed to measure the level of TGF-*β*1 in plasma in affected individuals compared to controls. They specifically investigated the involvement of five common single nucleotide polymorphisms (SNP) in TGF-*β*1 with the risk of development of keloid disease and HTS. 60 patients were studied, 15 with HTS and 45 with keloid disease, and 18 controls where enzyme-linked immunosorbent assay (ELISA) was used. A polymerase chain reaction-restriction fragment length polymorphism technique was selected for genotyping TGF-*β*1 polymorphisms. 133 patients provided DNA samples (101 keloid, 32 HTS) and 200 control samples were analyzed. The patients and controls were Caucasians originating from Northern Europe. Results illustrate no statistically significant difference in the plasma levels of TGF-*β*1 between patients with HTS, keloid disease, and controls. A statistically significant difference could not be detected in genotype or allele frequency distributions among patients and controls for codons 10, 25, and 263 and for −509 and -800 TGF-*β*1 gene SNPs [51].

Nakashima et al. performed a two-stage genome-wide association study (GWAS) of 517 patients and 2,385 controls in the Japanese populace. They identified four SNPs in three chromosomal regions. The most noteworthy correlation was in the region rs873549 on chromosome 1q41 fine mapping of a 125 kb region using 44 SNPs revealed that a 40 kb LD block could include critical genetic alteration(s) for susceptibility to keloid; however, no protein-coding gene in this region is known. For the second most significant association to rs1511412 at 3q22.3, fine mapping using 43 SNPs inside a 650 kb interval was conducted. This interval included two genes, namely, *LOC389151*, a hypothetical gene placed 24 kb telomeric to rs1511412, and *FOXL2*, which is positioned 47 kb centromeric to rs1511412. Lastly, there was rs8032158 at 15p21.3 and fine mapping of this 250 kb interval using 33 SNPs showed that the key region of involvement with keloid susceptibility includes *NEDD4* [56].

Brown et al. investigated the role of the major histocompatibility complex (MHC), also called the human leukocyte antigen (HLA) system, in keloid etiology within the Afro-Caribbean population. Multiple reports have found that hypertrophic scars and keloids have immune cell infiltrates into the scars, and these contain various immune cells, including elevated levels of HLA-DR and CD1a, which point to the direction of involving the MHC complex in these responses [57–59]. Brown et al. studied the HLA-DRB1 alleles in all of the participants by using a semiautomated typing system of reverse hybridization PCR utilizing sequence-specific oligonucleotide probes. With regards to phenotype frequencies, the most prominent variations were for alleles HLA-DRB1^*∗*^04, ^*∗*^09, ^*∗*^12, and ^*∗*^15. HLA-DRB1^*∗*^15 appeared to be associated with a susceptibility to keloid disease; however, this was not statistically significant [60]. The observations from the above gene association studies are summarized in Table 3.

## 4. Transcriptomics

Transcriptomics is the study of the transcriptome,which is a set of RNA transcripts that are produced by the genome. Gene expression is determined by analyzing the mRNA levels of the genes being studied. Traditionally, this is done using in-situ hybridization or northern blots. However, these methods are specific to only a few genes in question and not broad-ranging. Reverse transcription-polymerase chain reaction (RT-PCR) and ribonuclease protection assays, although more sensitive in detection, are also limited to a few genes in question. With the introduction of microarray technologies and next-generation sequencing, the landscape for high-throughput transcriptomics assays significantly changed. Microarray technology is a powerful chip-based tool which allows for great amounts of functional information to be gathered. Microarrays of complementary DNA (cDNA) sequences allow hybridization-based expression analysis of many genes [61].

A study by Chen et al. aimed to study the differences in gene expressions between keloids and normal skin using cDNA microarray from 6 individuals, 3 keloid and 3 normal [62], Table 4. They evaluated gene expression in over 8,400 human genes. 402 genes (4.79%) had variance in expression between keloids and normal samples; 152 genes were under-expressed, and there was overexpression of 250 genes. Collagen genes expression was enhanced in keloids compared with normal skin. The −1 chain of collagen XI was upregulated compared to any other gene in keloids. Upregulated genes in keloids compared to healthy skin included *α* − 2 chain of collagen Type 5, pro (*α* − 1) chain of collagen Type 3, pro (*α* − 2) chain of collagen Type 1, *α* − 1 chain of collagen Type 4, fibronectin, and a few proteoglycans. Additionally, the gene for collagenase inhibition was upregulated in keloids compared to normal skin. However, the gene expression variation of the tissue inhibitors of metalloproteinases was not significant when normal skin was compared to keloid scar. Gene expression for nerve growth factor (NGF) and TGF-*β*1 was considerably elevated in keloids when compared to normal skin. Furthermore, the gene expression of the VEGF165 receptor was reduced in keloids. Compared with keloids, gene expression of ASK1, KIAA0018, and p21 (could stimulate cellular apoptosis) was overexpressed, yet, in normal skin, the inhibited-apoptosis gene of secreted apoptosis-related protein 1 (SARP1) was under expressed [62].

Shih and Bayat used publicly available data on genes involved in keloids and formulated a list of 25 genes that were dysregulated in keloids [63], Table 4. These 25 genes were either up or down regulated in more than one microarray analysis. The David Bioinformatic Functional Annotation Tool was used to annotate the 25 genes. 12 out of the 25 genes were extracellular matrix genes, these included aggrecan (ACAN), fibronectin 1 (FN1), osteoglycin (OGN), collagen type I alpha 1 (COL1A1), collagen type I alpha 2 (COL1A2), collagen type IV alpha 2 (COL4A2), collagen type V alpha 2 (COL5A2), collagen type VI alpha 1 (COL6A1), collagen type XV alpha 1 (COL15A1), periostin osteoblast specific factor (POSTN), TGF-*β*RIII, and versican (VCAN). Out of 25 genes, 8 of them were related to inflammation pathways, cytokines or immune-regulation, comprising of FN1, EGFR, insulin-like growth factor binding protein 7 (IGFBP7), alpha-2-macroglobulin (A2M), ACAN, annexin A1 (ANXA1), TGF-*β*RIII, and VCAN. 5 out of the 25 genes involved in apoptosis, including ANXA1, EGFR, FN1, hypoxia-inducible factor 1 alpha subunit (HIF-1A), and Serpin peptidase inhibitor clade F (SERPINF1) [63].

Since keloids and hypertrophic scars are distinguished based on highly dense collagen fibers, Freidman et al. investigated the ratio of type I/III collagen in normal skin, normal scars, keloids and hypertrophic scars [39]. Their data illustrates that there is a significant elevation of type I to type III collagen in keloids compared to the other tissues. Freidman et al. also analyzed mRNA normal state levels coding for *α*1 (I) procollagen through northern blot analysis using *α*1 (I) procollagen and *γ* actin cDNA probes. They report that in keloids there is a distinct increase in *α*1 (I) procollagen mRNA. This elevation in *α*1 (I) procollagen mRNA in keloids resulted from higher expression of the gene expressions due to a considerably higher transcription rate of the *α*1 (I) procollagen gene in keloids, as determined by nuclear run-off transcription. In hypertrophic scars, the transcription rate of the *α* (I) procollagen gene was also elevated, however there was no rise in *α*1 (I) procollagen mRNA level or change in the type I/III collagen ratio in hypertrophic scars. This paper therefore shows that the rate of gene transcription of *α*1 (I) procollagen is high in both keloids and hypertrophic scars, however keloids display normal state levels of *α*1 (I) procollagen mRNA and simultaneous increases in type I collagen. The data here indicate that pretranscriptional and posttranscriptional regulation dictates type I collagen synthesis. In hypertrophic scars, even though the rate of transcription is high for *α* (I) procollagen gene, transcriptional modifications decrease the levels of mRNA coding for *α*1 (I) procollagen [39].

Hahn et al. aimed to shed light on keratinocytes and fibroblasts and their role in keloid scarring [64], Table 4. They conducted genomic profiling of keloid-derived cells and conducted bioinformatics analyses. Keloids harvested from different regions of the body from both females and males and from individuals from various ethnicities such as African American and Caucasian were used for this study. Cells were derived from nine keloids, four nearby nonlesional keloid patient skin samples, and three normal skin samples were analyzed using expression microarrays. Next, RNA was removed, labeled and hybridized to Affymetrix Human Gene 1.0 ST microarray chips. Interestingly, a large number of genes that were higher in keloid cells compared with normal cells were also elevated in cells that were harvested from nonlesional skin beside the keloids. One gene that was significantly decreased in fibroblasts from the keloids when compared to nonlesional normal fibroblasts is cannabinoid receptor 1 (CNR1) [64]. This gene codes for one of two G-protein coupled receptors for cannabinoids, a cluster of lipids that includes D9-tetrahydrocannabinol in addition to endogenous ligands, or endocannabinoids. An analysis of the pathways involving the fibroblast gene set revealed that the molecular function “transcription regulatory activity” was greatly significant, with 27 genes up or downregulated in keloid as compared to normal fibroblasts, including 15 HOX genes [64]. Also, “organ morphogenesis” was a highly significant biological process correlated to this gene set, followed by “regulation of cell proliferation”. Other important pathways related to the keloid fibroblast gene set were “O‐glycan biosynthesis” and “altered canonical Wnt signaling” [64].

In keratinocytes, there was a small subset of genes that were elevated in keloid compared to nonlesional or normal cells. These include the differentially expressed histone cluster 1 genes between keloid and normal cells: HIST1H1A, HIST1H1B, HIST1H2BH, and HIST1H4F [64]. In addition, elevated in keloid keratinocytes were the genes coding for the histone chaperone ASF1 antisilencing function 1 homolog B (ASF1B) and centromere proteins K and H. The highly expressed genes playing a role in chromatin organization and kinetochore structure indicate higher mitotic rates in keloid keratinocytes. Hahn et al. conducted genomic profiling of keloid keratinocytes, which uncovered an array of genes in the gene set encoding various types of proteins. However, a common trend was that a lot of these genes were previously implicated in cell adhesion, differentiation, migration, invasion, and epithelial to mesenchymal transition (EMT). The hyaluronan synthase 2 (HAS2) gene, encoding one of three HAS enzymes involved in biosynthesis of hyaluronan, an important part of the epidermal ECM was 4.38‐fold increased in keratinocytes from keloid. Elevated expression of HAS2 has been linked to cancer cell proliferation and metastasis, and overexpression subsequently led to higher keratinocyte migration in vitro. In keloids Keratin 7 (KRT7) was increased 3.52‐fold compared to normal keratinocytes. KRT7 is a low molecular weight type II keratin that is expressed in simple epithelial tissues, which includes glandular epithelia, but not expressed in normal epidermal keratinocytes. KRT7 expression was also discovered in a subgroup of colorectal cancers. In keloid keratinocytes, Lysyl oxidase (LOXL2) was enhanced compared with normal keratinocytes. LOXL2 is important for the formation of crosslinks in collagen and elastin. LOXL2 has been associated with different processes in cancer progression, for example, EMT, tumor cell invasion, tumor progression, and regulation of epidermal differentiation. In line with diminished differentiation, keloid keratinocytes presented lowered expression of KRT1 and KRT16; cadherin‐11 (CDH11) and vimentin (VIM) were both increased in keratinocytes from keloids [64], Table 4.

The Smads pathway plays a critical role in regulating collagen synthesis, and Smads are involved in TGF-*β*-dependent stimulation [67]. It has been reported that TGF-*β* is involved in initiating and maintaining fibroblast activation [55, 67]. Furthermore, TGF-*β* promotes the production of collagen and supports wound healing through the regulation of fibroblast growth, differentiation and proliferation [68]. Wang et al. investigated the loss of Smad3 function and studied how this affects abnormal TGF-*β* signal transduction in fibroblast cells of keloids by utilizing synthetic siRNA. They transiently infected keloid fibroblasts with siRNA−1, −2, and −3 to knock down Smad. Since an overabundance of collagen synthesis is a tell-tale sign of keloids, they evaluated types I and III procollagen expression after the downregulation of Smad3 by siRNA-3. Results showed that cells transfected with Smad3 siRNA-3 led to a significant reduction in mRNA levels of types I and III procollagen. In addition to types I and III procollagen expression, they also identified a significant lowering in the mRNA level of TGF-*β*1 [69].

Wu et al. investigated hypertrophic scar gene expression [65], Table 4. They selected five samples of early human postburn HTS and isolated RNA for cDNA microarray analysis. In total, 97 differentially expressed genes were identified in all of the five samples. Of these 97 genes, 94 genes related to scars were upregulated and 3 of the genes were downregulated. These genes that were differentially expressed represented different cellular processes, such as genes that are proto-oncogenes, tumor suppression genes, genes related to immunity, cell signaling, metabolism, cytoskeleton, apoptosis, translation, transcription, and some genes which were unidentified. Five of the upregulated cytoskeletal genes were further examined through in-situ hybridization in HTS 3, 6, 9, and 12 months old; normal scar; and healthy skin. Results demonstrated that the expression of profilin, *α*-smooth muscle actin, vimentin, tropomyosin TM30, and BM40 mRNA was substantially high in early postburn HTS in the samples from 3 month old scars, more specifically the expression of *α*-smooth muscle actin. Postremodeling of HTS, the expression of these genes decreased rapidly. The most highly expressed gene was *α*-smooth muscle actin at the level of mRNA. Subsequently, Wu et al. discovered *α*-smooth muscle actin protein in postburn hypertrophic scars that were 3, 6, 9, and 12 months old; normal scars; and healthy skin through immunohistochemistry analysis. Results illustrate that *α*-smooth muscle actin protein was primarily present in the initial phase of hypertrophic scarring [65].

Dasu et al. cultured fibroblasts from hypertrophic scars and normal skin and compared 12,625 genes in the HTS fibroblasts to normal fibroblasts [66], Table 4. They illustrated the elevated expression of 12 genes in comparison to fibroblasts from normal skin, while 14 genes' expression decreased. Some of the top upregulated genes were quinoline phosphoribosyltransferase, serine (or cysteine) proteinase inhibitor clade B, collagen type XIII *α*-1, plasminogen activator urokinase, and neuromedin. Some of the downregulated genes were pregnancy specific *β* glycoprotein, stromal cell derived factor-1, serine protease 23, tubulin-*α*-1, and cardiac actin gene [66].

## 5. Proteomics

Ong et al. aimed to detect proteins which had varied expression in keloid skin compared to normal skin tissue using two‐dimensional gel electrophoresis with computer‐aided image analysis and mass spectrometric procedures [70]. The results from this study are summarized in Table 5. In normal skin, the spots uncovered a mean of 598 resolved protein spots. Thirty‐five protein spots were then isolated for mass spectrometric analysis, which was correlated to 23 different proteins and isoforms. In keloid scars, spot detection revealed a mean of 902 resolved protein spots. Forty‐four protein spots were removed for mass spectrometric analysis, which correlated to 32 different isoforms and proteins. Most of the differential proteins expressed were not seen in both normal and keloid skin. The proteins identified in normal skin were categorized. For example, an energy metabolism protein was triosephosphate isomerase 1; protein synthesis, folding, and degradation proteins were heat shock protein 27, heat shock 70 kDa protein 8 isoform 2 variant, eukaryotic translation initiation factor 5A, and alpha crystalline B chain. Cytoskeleton proteins were keratin 10: intermediate filament, keratin 1: intermediate filament, keratin type II cytoskeletal 2 epidermal: proline arginine‐rich end leucine‐rich repeat protein precursor and intermediate filament were also detected. Physiological proteins; carbonic anhydrase II mutant E117q holo form, carbonic anhydrase 1, carbonic anhydrase II mutant E117q holo form, transferrin: transport of iron, calmodulin 3, haptoglobin Hp2, chain D deoxy Rhb1.1, alpha 1‐antitrypsin, and fibrinogen beta chain. Tumor suppressor proteins such as gelsolin isoform *α* and *β*. Antifibrogenesis protein manganese superoxide dismutase, immunity related proteins such as complement component 3 and immunoglobulin kappa chain V–III region B6 were also detected [70].

In the keloid scar tissue, proteins identified were cytoskeleton proteins such as Tropomyosin 1 alpha chain isoform 5, smooth muscle and nonmuscle myosin alkali light chain isoform 1, profilin 1, rho GDP dissociation inhibitor alpha, actin‐related protein 2 isoform *b*, gelsolin‐like capping protein, and transgelin (SM22). Extracellular matrix proteins such as Alpha 1 type I collagen preprotein, carboxy‐propeptide of alpha 1 (III) procollagen, alpha‐1 type III collagen, type I collagen and collagen type V alpha 1. Physiological proteins such as ferritin heavy chain, light chain, ferritin heavy polypeptide 1, hemoglobin‐delta, chloride intracellular channel 1, fibrinogen gamma chain, serum albumin precursor, and ras‐related nuclear protein (RAN). Inflammation associated proteins such as S100A4, S100A8, S100A10, and S100A9. Antioxidant proteins such as thioredoxin/thiol‐redox, peroxiredoxin I. Fibrogenesis proteins such as mast cell tryptase *β* III, chain D^#^ human *β*‐tryptase, chain D^#^ human *β* II, macrophage migration inhibitory factor (MIF) chain C. Antifibrosis proteins such as pigment epithelium‐derived factor, asporin. Tumor suppressor proteins such as galectin‐1 chain A, stratifin (14‐3‐3 sigma), maspin (serine/cysteine proteinase inhibitor). Protein synthesis, folding and degradation proteins such as, eukaryotic translation initiation factor 5A isoform I variant A (eIF5A‐1) [70].

Tan et al. utilized proteomics approaches to identify differentially expressed proteins in hypertrophic scars in comparison to healthy skin using Isobaric Tags for Relative and Absolute Quantitation (iTRAQ) labeling equipment [71]. The results from this study are compiled in Table 6. Next, they performed high-throughput 2D LC-MS/MS to understand the relative quantitative variance in the expression of proteins between healthy skin and hypertrophic scar tissue. Tissue samples isolated from three patients revealed 3166 proteins that were screened by iTRAQ. Forty-one of those proteins were upregulated and associated with extracellular matrix, and 48 were downregulated proteins that were engaged in dynamic junction and structural molecule activity. Some examples of top upregulated proteins were cartilage oligomeric matrix protein, fibromodulin, aspirin, pleiotrophin and collagen Type XII, and alpha 1 to name a few. Some of the downregulated proteins were pyruvate kinase muscle 2, inner membrane protein, malate dehydrogenase 2, dynein cytoplasmic 1, NAD (mitochondrial), mitochondrial (mitofilin), calpastatin, heavy chain 1, and integrin beta 4 to name a few. Within the differentially expressed proteins, 20 of 41 upregulated proteins were evaluated, utilizing cytoscape software to reveal a diverse functional network. BinGO enrichment showed that 20 upregulated protein classifications were higher in the biological process, cellular component, and molecular function groups. Amid these proteins, extracellular matrices such as collagen, type I, alpha 1 (COL1A1); collagen, type I, alpha 2 (COL1A2); collagen, type III, alpha 1 (COL3A1); collagen, type V, alpha 1 (COL5A1), cartilage oligomeric matrix protein (COMP), fibronectin 1 (FN1), thrombospondin 1 (THBS1), and tenascin C (TNC) were recognized to be the highest cluster. The majority of downregulated proteins, including EVPL, JUP, DSC3, DSP, and PPL, were particular to the epidermis and played a role in the cell junction [71].

A multitude of peptides, such as recombinant human IL10-RGD (rhIL10-RGD), transforming growth factor beta peptide antagonists, and P144 peptide, have been thoroughly studied in the treatment of hypertrophic scar [72, 73]. However, in hypertrophic scar pathogenesis, the part played by endogenous peptides has seldom been studied. Li et al. conducted a comparative peptidomics study of human hypertrophic scar tissue with matched normal skin to investigate how endogenous peptides are involved in scar formation by using liquid chromatography tandem mass spectrometry (LC-MS/MS) [72]. Using LC-MS/MS, Li et al. found a total of 1,697 nonredundant peptides in hypertrophic scar tissues and the matched normal skin. 179 peptides had varied expression, which was significant in the hypertrophic scar group in comparison to the normal skin group, with 95 upregulated peptides and 84 downregulated peptides. Twenty-two peptide fragments from the Vimentin proteinwere comprised of the highest amount of the endogenous peptides. Precursor proteins hemoglobin subunit alpha, hemoglobin subunit beta, histone H1.4, neuroblast differentiation-associated protein AHNAK, and hemoglobin subunit delta gave rise to other peptides. GO and pathway analysis techniques were used to study the functions of the differentially expressed peptides and their precursor proteins [72]. Enrichment analysis demonstrated that cellular process, biological regulation, cell, cell part binding, and structural molecule activity encompass highly involved GO classes. Analysis of the pathways demonstrated that the majority of precursor proteins were involved in pathways involving ribosomes, transcriptional dysregulation in cancer, and the PPAR signaling pathway. The peptides were compared against a database of functional peptides from BIOPEP, PeptideDB, CAMP, and APD2. Of the 179 peptides found, 78 shared homology with antimicrobial peptides. Furthermore, five matched known immunomodulatory peptides were predicted with a webserver. The elevated overlap of sequences suggests the likely function of these identified peptides [72].

### 5.1. Metabolomics

Metabolomics is known as the large-scale study of metabolites such as small molecules within cells, biofluids, tissues, or organisms. Jointly, these small molecules in a biological system and how they interact is known as the metabolome.

Keloids and HTS are fibroproliferative disorders with mechanical stress being associated with scar growth. Furthermore, a keloid is described as a state of chronic inflammation. Literature suggests that this lengthy, active inflammatory state is a result of cyclical skin tension, which activates different mechanotransduction pathways. Akaishi et al. suggested the “neurogenic hypothesis” which proposes that mechanical stress raises neuropeptides within the skin, which leads to neurogenic inflammation [74]. Neurogenic inflammation is facilitated by the discharge of neuropeptides from sensory endings. Furthermore, these neuropeptides may upregulate the gene expression of growth factors such as TGF-*β* and NGF in different such as fibroblasts within the dermis. Sensory nerves which terminate in the skin often contact epidermal and dermal cells, and these nerves secrete neuropetides which can directly regulate the cellular function of fibroblasts, mast cells, keratinocytes, Langerhans cells, dermal microvascular endothelial cells, and infiltrating immune cells [75]. Amongst these neuropeptides, neurokinin A (NKA), substance P (SP), calcitonin gene-related peptide (CGRP), vasoactive intestinal peptide (VIP), and somatostatin (SOM) collectively affect the function of skin and immune cells, for example: cell proliferation, sensory neurotransmission, cytokine production, presentation of antigens, degradation of mast cells, dilation of blood vessels, and elevation in vascular permeability under physiological or pathophysiological conditions [75, 76]. These proinflammatory responses cause neurogenic inflammation [74].

Investigation into the changes in lipid profile and the functional roles of the metabolism of lipids in keloids has been conducted to detect target molecules with the purpose of designing novel therapeutic interventions. Recently, it has been suggested that another causal factor for keloid formation is altered lipid metabolism, more specifically the metabolic processes that correlate with essential fatty acids. There is a possibility that these deviations may promote the inflammatory reaction in keloids. In keloids, as opposed to normal skin, the levels of the metabolic products of the arachidonic acid (AA) and eicosapentaenoic acid (EPA) cascades are altered [77]. It is also probable that an imbalance between the proinflammatory prostaglandins (PGs) and leukotriene (LTs) and the antiinflammatory lipoxin (LXs), protectin D (PDs), and resolvin (Rvs) within these cascades leads to inflammation [77]. Lipids also serve as pools of secondary messengers such as diacylglycerol (DAG) and arachidonic acid (AA) that promote the proliferation of fibroblasts [77].

## 6. Epigenomics

Epigenetics is the study of the heritable changes in a gene, which take place without the DNA nucleotide sequence changing [78]. There is evidence that changes in the chromatin structure of DNA (instead of the nucleotide sequence) are associated with heritable changes in gene expression [78]. For example, stimuli from the environment such as drugs, toxins, and nutrients can all affect specific gene expression without modifying the genetic code. Epigenomic systems include DNA methylation and covalent posttranslational modifications of histones. Chemical modifications of histones, include methylation, N-terminal acetylation, ubiquitination, and phosphorylation [78].

### 6.1. DNA Methylation

Since a keloid is a benign tumor-like mass and has a similar process to tumorigenesis, Jones aimed to establish whether abnormal methylation of genes, a phenomenon seen in tumorigenesis, also occurs in keloids [79]. There are two forms of abnormal methylation patterns observed in cancer cells. The first is gene-specific hypermethylation, which occurs on the CpG islands in the promoter regions of genes, typically causing a reduced expression of the gene. The second form is genome-wide hypomethylation [80]. Jones performed genome-wide profiling utilizing the Infinium HumanMethylation450 BeadChip program to study differentially methylated genes from 6 keloid and 6 normal skin samples. They developed a 3-tiered approach to study CpGs. This research concentrated on the promoter region CpGs; hence, just Tier 3 CpGs were correlated for associated genes. Tier 3 yielded 685 differentially methylated CpGs. Of the CpGs at Tier 3, 175 were hypermethylated and 510 were hypomethylated with 190 CpGs (28%) in the promoter and 495 (72%) in nonpromoter regions. Of the 190 promoter region CpGs, 128 were hypomethylated and 62 were hypermethylated [79].

### 6.2. Histone Modification

Histone deacetylases (HDACs) remove acetyl groups from histones and histone acetyltransferases (HATs) add acetyl groups to histones. These are epigenetic modifying enzymes which affect gene expression (acetylation promotes transcription) [81]. Fitzgerald O'Connor et al. aimed to establish the expression profiles of specific HDACs in normal tissue and keloids [82]. They used immunohistochemistry to assess three types of skin tissue: normal healthy skin from humans, normal scar tissue harvested from patients taking part in melanoma reexcision and keloid scars that are older than 6 months. Tissue sections were examined for HDAC1, HDAC2, HDAC4, and HDAC7. Scar-related fibroblasts from both normal and keloid scars displayed a significant elevation of HDAC2, but not HDAC1, 4, or 7. Epidermal HDAC expression was the same in scarred regions compared to nonscarred regions. HDAC2 was upregulated in tissue from scars, and this result has been validated by studying a wound repair mouse model [82].

### 6.3. MicroRNA

MicroRNAs (miRNAs) are small noncoding RNAs that can control protein expression in a negative manner. There are reports which state that miRNAs play a significant part in tissue fibrosis and the metabolism of ECM. However, in keloids, the regulation and function of miRNAs have not been thoroughly investigated. Kashiyama et al. aimed to detect miRNAs participating in keloid pathogenesis [83]. They conducted miRNA microarray analysis to compare miRNA expression profiles between fibroblasts from keloids (KFs) and normal fibroblasts (NFs). Three samples of fibroblasts from keloid and normal skin were analyzed and a sum of 27 miRNAs were discovered to be differentially expressed in KFs in comparison to NFs, out of these, there were 7 overexpressed miRNAs and 20 under-expressed miRNAs. From these miRNAs, miRNA-142-3p, and miR-196a demonstrated the largest fold changes (miR-142-3p: 9.856-fold and miR-196a: 0.093-fold in KFs as opposed to NFs) [83]. These two miRNAs were then further investigated. Kashiyama et al. established that the effect of passage and environmental change may affect miRNA expression in KFs. The amount of change was reported to be dependent on each individual miRNA. Therefore at passage 0 and 2, the KFs and NFs were compared by TaqMan real-time reverse transcriptase–PCR (RT-PCR) assay. Interestingly, the variance in miR-142-3p expression at passage 0 was completely lost at passage 2. In contrast, the difference in miR-196a tended to be increased postpassage 2. Hence, this data indicates that the expression of miRNA can be rapidly altered during early passages. To determine the processes through which these miRNAs support keloid pathology, three Internet databases, miRanda, TargetScan, and PicTar, were used to study potential target mRNAs of these miRNAs. For the miR142-3p, 2,634 genes in miRanda, 250 genes in TargetScanand 200 genes in PicTar were predicted. Among the three databases, 70 genes were common to all the databases. Some of the candidate genes examined were: ADCY9, BCLAF1, CFL2, COL24A1, HMGA2, and ROCK2. These genes are involved in collagen synthesis, apoptosis, actin filaments, collagen, transforming growth factor-*β* signaling, and Rho-actin signaling, respectively [83]. However, it should be noted that Kashiyama et al. were not able to distinguish variation in any of these candidate genes between samples from the nine KF and nine NF. Within the predicted target genes of miR-196a, they focused on collagens (COL1A1, COL1A2, and COL3A1) and they validated the elevated level of COL1A1, COL1A2, and COL3A1 mRNAs in their samples [83]. Furthermore, there is potential miR-196a target site in COL1A1, COL1A2, and COL3A1 in their 3′ untranslated region (UTR).

Li et al. also investigated miRNA expression profiles comparing keloid-derived fibroblasts and normal fibroblasts [84]. They performed in-depth miRNA profiling and a comparative miRNA analysis. Nine miRNAs were established to have varied expression levels between keloid and normal fibroblasts; six of these were significantly upregulated in KFs, including miR-30a-5p, miR-31-5p, miR-152, miR-23b-3p, miR-320c, and hsv1-miR-H7, and three of them were substantially downregulated. These included miR-143-3p, miR-4328, and miR-145-5p [84]. The largest fold change was exhibited in miRNA-145-5p, miR-4328, and miR-143-3p (miR-145-5p: 9.79 folds, miR-4328 : 7.61 folds, and miR-143-3p: 0.32 fold in KFs when compared to adult dermal fibroblasts). The bioinformatics analysis of the abovementioned differentially expressed miRNA targets showed the enrichment of these in multiple signaling pathways critical for wound healing of scars. TargetScan Human 5.1, DIANA-microT v3.0, and MicroCosmTargets version 5 are three databases that are publicly available that were used for miRNA target gene prediction, and the gene was identified as a potential target if it was predicted by all three databases. This research study revealed that many of the targets played important roles in multiple critical pathways linked to keloids, including transforming growth factor-*β* (TGF-*β*) family and mitogen-activated protein kinase (MAPK) pathways, apoptosis, and the cell-cycle pathways [84].

## 7. Conclusion

The term “omics” in biology refers to studies of biological molecules on a comprehensive and global level [85]. For example, the study of genomics analyses genetic variants in disease across millions of genes. On the other hand, genetics studies a single gene in depth [85]. Omics approaches share three key characteristics in comparison to conventional methods. The first characteristic of omics, unlike traditional techniques, is that omics utilizes high-throughput, data-driven and top-down methods. Second, in omics, the aim is to understand the cell as one ‘integrated system' as opposed to sets of distinct parts by utilizing data regarding the relationships between many molecular entities [86]. The third characteristic is that the high-throughput ‘omics' approach generates a large quantity of data, the evaluation of which necessitates statistical and computational efforts [87]. As presented in this review paper, omics approaches are used in the fields of genomics, transcriptomics, proteomics, epigenomics, and metabolomics, amongst others. A combinatorial study of different omics approaches is more effective in studying a complex medical condition such as keloid and hypertrophic scars as opposed to one omics approach, which would present data of a single type.

**Figure 1:**
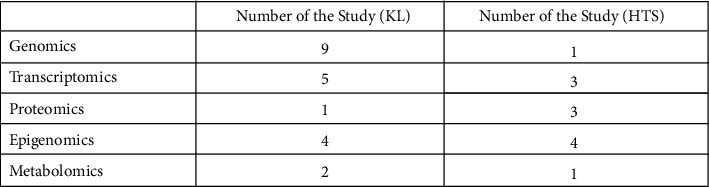
Summary of the omics studies represented in this review paper pertaining to Keloids and HTS.

In this review paper, we start with a discussion of the steps which occur in normal wound healing and then investigate abnormal wound healing before we delve into keloids and hypertrophic scar formation. Since excessive scarring and abnormal wounds can occur due to dysregulation at the level of the genome, transcription, translation or even through epigenetic factors, we decided to review papers discussing genomics, transcriptomics, proteomics, epigenomics, and metabolomics with regards to keloids and HTS. In our review of scar genomics, 9 of the papers discuss keloids and 1 paper discusses HTS. For transcriptomics, 5 papers discuss keloids and 3 discuss HTS. For proteomics, 1 paper focuses on keloids and 3 papers focus on HTS. For epigenomics, 4 papers focus on keloids, and 4 focus on HTS. Finally, for metabolomics, 2 papers are on keloids and 1 paper is on HTS. This summary of papers is presented in Figure 1.

## 8. Treatment

Omics approaches are the next-generation of precision medicine for the treatment of keloids and hypertrophic scars. Precision medicine considers both individual variability and population characteristics in an effort to deliver healthcare that is tailored to each individual; this technique broadens biological understanding and explores the biological diversity amongst people [88]. Personalized medicine is based heavily on biomarker-driven strategies, such as genes, transcripts, metabolites, and proteins. Therefore, omics approaches to studying abnormal scars provide high-throughput technologies which gives a global view of an individual's biological system. Omics provides a way to predict normal and pathological states based on an integrative understanding of the genetic environment all the way down to the molecules, on both an individual and population level. Since omics data provides multi-dimensional information, mathematical modeling is critical to building classifiers for successful medical decision-making [89]. In addition, machine learning and chemometric methods are required to gain insights from the dataset [90].

In this review, we are at the beginning of the discussion on omics on keloids and hypertrophic scars. As this field grows with developments in technology for omics evaluation, we hope to continue our investigation of the omics of pathological scarring such as keloids and HTS. Keloids and HTS affect millions of individuals worldwide and can often cause significant pain and discomfort, be aesthetically displeasing and diminish the quality of one's life; hence, we look forward to advancement in knowledge within this field.

## Figures and Tables

**Table 1:** Inheritance patterns observed through pedigree analysis.

Study	Inheritance type	Penetrance	Population studied
[47]	Autosomal dominant	Incomplete clinical	African, American
		penetrance	Caucasian
		Variable expression	Japanese
			African, Caribbean
[34]	Autosomal recessive	N/A	Igbo dora people of west Nigeria
[48]	Autosomal dominant	Incomplete clinical penetrance	Han Chinese population

**Table 2:** Summary of linkage studies.

Chromosomal locus	Linkage to genes within this locus
2q23 [52]	TNFAIP6 [52]
7p11 [52]	EGF receptor [52]
18q21.1 [53]	SMAD2, SMAD4, SMAD7, and PLAS2 [53]
10q23.31 [54]	FAS [54]

**Table 3:** Gene association for keloid and hypertrophic scars in different populations.

Gene association	Observations
Codons 10, 25, and 263 and for −509 and −800 single nucleotide polymorphisms of the TGF-*β*1 gene	Keloid/HTS patients and controls for codons were not statistically significant for genotype or allele occurrence [51]
Region rs873549 on chromosome 1q41	Keloid only [56].
rs1511412 at 3q22.3	Keloid only. *LOC389151*, a hypothetical gene located 24 kb telomeric to rs1511412, and *FOXL2* gene association [56].
rs8032158 at 15p21.3	Keloid only. *NEDD4* gene association [56].
HLA-DRB1^*∗*^04, ^*∗*^09, ^*∗*^12 and ^*∗*^15. HLA-DRB1^*∗*^15	Predisposition to keloids [60]

**Table 4:** Transcriptomic study of differentially regulated genes in keloids, hypertrophic scars, and normal skin.

Gene	Keloids	Hypertrophic scars	Normal skin
−1 chain of collagen XI	↑ [62]	⟶	
*α* − 2 chain of collagen type 5	↑ [62]	⟶	
Pro (*α* − 1) chain of collagen type 3	↑ [62]	⟶	
Pro (*α* − 2) chain of collagen type 1	↑ [62]	⟶	
*α* − 1 chain of collagen type 4	↑ [62]	⟶	
Fibronectin	↑ [62]	⟶	
Nerve growth factor (NGF)	↑ [62]	⟶	
TGF-*β*1	↑ [62]	⟶	
VEGF165 receptor	↓ [62]	⟶	
ASK1		⟶	↑ compared to KL
KIAA0018		⟶	↑ compared to KL
p21		⟶	↓ compared to KL
ACAN	↑ [63]	⟶	
COL1A1	↑ [63]	⟶	
COL1A2	↑ [63]	⟶	
COL4A2	↑ [63]	⟶	
COL5A2	↑ [63]	⟶	
COL6A1	↑ [63]	⟶	
COL15A1	↑ [63]	⟶	
FN1	↑ [63]	⟶	
OGN	↑ [63]	⟶	
POSTN	↑ [63]	⟶	
TGF-*β*riii	↓ [63]	⟶	
VCAN	↑ [63]	⟶	
ANXA1	↑ [63]	⟶	
EGFR	↓ [63]	⟶	
HIF-1A	↑ [63]	⟶	
SERPINF1	↓ [63]	⟶	
CNR1	↓ [64]	⟶	
HOXA5, HOXA7, HOXC11	↓ [64]	⟶	
HIST1H1A, HIST1H1B, HIST1H2BH, and HIST1H4F	↑ [64]	⟶	
ASF1B	↑ [64]	⟶	
Proteins K and H	↑ [64]	⟶	
HAS2	↑ [64]	⟶	
KRT7	↑ [64]	⟶	
LOXL2	↑ [64]	⟶	
KRT1	↓ [64]	⟶	
KRT16	↓ [64]	⟶	
CDH11	↑ [64]	⟶	
VIM	↑ [64]	⟶	
*α*-smooth muscle actin	⟶	↑ [65]	
Tropomyosin TM30	⟶	↑ [65]	
Vimentin	⟶	↑ [65]	
Profilin	⟶	↑ [65]	
BM40	⟶	↑ [65]	
Quinoline phosphoribosyltransferase	⟶	↑ [66]	
Serine (or cysteine) proteinase inhibitor clade B	⟶	↑ [66]	
Collagen type XIII *α* − 1	⟶	↑ [66]	
Plasminogen activator urokinase	⟶	↑ [66]	
Neuromedin	⟶	↑ [66]	
Stromal cell derived factor −1, serine protease 23	⟶	↓ [66]	
Pregnancy specific *β* glycoprotein 7	⟶	↓ [66]	
Tubulin-*α* − 1	⟶	↓ [66]	
Cardiac actin gene	⟶	↓ [66]	

**Table 5:** Proteins observed in normal skin, keloid tissue and in both normal and keloid tissue using 2D Gel Electrophoresis [70].

Proteins observed only in normal skin [70]	Proteins observed only in keloids [70]	Proteins observed in both normal skin and keloids [70]

Triosephosphate isomerase 1	Tropomyosin 1 alpha chain isoform 5	Alpha 1‐antitrypsin
Heat shock protein 27	Smooth muscle and nonmuscle myosin alkali light chain isoform 1	Chloride intracellular channel 1
Heat shock 70 kDa protein 8 isoform 2 variant	Profilin 1	Proline arginine‐rich end leucine‐rich repeat protein precursor
Eukaryotic translation initiation factor 5A	Rho GDP dissociation inhibitor alpha	Carbonic anhydrase 1
Alpha crystalline B chain	Actin‐related protein 2 isoform b	Fibrinogen gamma chain
Keratin 10	Gelsolin‐like capping protein	S100A10
Keratin 1	Transgelin (SM22)	Chain D^#^ human *β*‐tryptase
Keratin type II cytoskeletal 2 epidermal	Alpha 1 type I collagen preproprotein	
	Carboxy‐propeptide of alpha 1 (III) procollagen	
Carbonic anhydrase II mutant E117q holo form	Alpha‐1 type III collagen	
Transferrin	Type I collagen	
Calmodulin 3	Collagen type V alpha 1	
Haptoglobin Hp2	Ferritin heavy chain	
Chain D deoxy Rhb1.1 (recombinant haemoglobin)	Ferritin heavy polypeptide 1	
Fibrinogen beta chain	Haemoglobin‐delta	
Gelsolin isoform b		
Gelsolin isoform a	Serum albumin precursor	
Prostatic binding protein	Ras‐related nuclear protein (RAN)	
Manganese superoxide dismutase	S100A4, S100A8,S100A9	
Complement component 3	Thioredoxin/thiol‐redox	
Immunoglobulin kappa chain V-III region B6	Peroxiredoxin I	
	Mast cell tryptase *β* III	
	Macrophage migration inhibitory factor (MIF) chain C	
	Pigment epithelium‐derived factor	
	Asporin	
	Galectin‐1 chain A:	
	Stratifin (14‐3‐3 sigma)	
	Maspin (serine/cysteine proteinase inhibitor)	
	Eukaryotic translation initiation factor 5A isoform I variant a (eIF5A‐1)	

**Table 6:** Comparison of upregulated and downregulated proteins in hypertrophic scars compared to normal skin using iTRAQ labeling [71].

Up regulated proteins [71]	Down regulated Proteins [71]

Cartilage oligomeric matrix protein	Inner membrane protein, mitochondrial (mitofilin)
Fibromodulin	Malate dehydrogenase 2, NAD (mitochondrial)
Asporin	Calpastatin
Pleiotrophin	Pyruvate kinase muscle 2
Collagen type XII alpha 1	Dynein, cytoplasmic 1, heavy chain 1
Tenomodulin	Integrin *β*4
Peptidylprolyl isomerase B (cyclophilin B)	Transketolase
Collagen, type V, alpha 1	H1 histone family, member 0
Thrombospondin 1	

## Data Availability

Previously reported data were used to support this study and are available in the reference list. These prior studies (and datasets) are also cited at relevant places within the text as references [1–90].
